# The predictive potential of surrogate indicators of insulin resistance for type 2 diabetic kidney disease

**DOI:** 10.3389/fendo.2026.1705701

**Published:** 2026-01-27

**Authors:** Qiuhui Tian, Yu Liu, Qiumei Cao, Meixu Zhang, Fengying Zhu, Yukai He, Xiaoling Zhu

**Affiliations:** 1 Department of Gastroenterology, Jiangjin Hospital, Chongqing University of Chinese Medicine, Chongqing, China; 2 Department of Gastroenterology, Chong Qing Jiangjin District Hospital of Chinese Medicine, Chongqing, China

**Keywords:** type 2 diabetes mellitus, diabetic kidney disease, insulin resistance, predictive biomarkers, metabolic dysfunction

## Abstract

**Background:**

Approximately 40% of patients with diabetes develop diabetic kidney disease (DKD), necessitating renal replacement therapy such as dialysis or transplantation. Furthermore, DKD significantly elevates the risk of cardiovascular events and all-cause mortality, while imposing a substantial economic burden on healthcare systems. This study investigates the association between surrogate markers of insulin resistance (IR)—the triglyceride-glucose (TyG) index, TyG-body mass index (TyG-BMI), and the triglyceride-to-high-density lipoprotein cholesterol ratio (TG/HDL-C)—and DKD in individuals with type 2 diabetes mellitus (T2DM), evaluating their predictive utility for DKD progression.

**Methods:**

We enrolled 311 patients diagnosed with T2DM between January 2024 and April 2025. Baseline clinical characteristics and variations in IR markers across proteinuria stages were analyzed. Multivariable logistic regression assessed the association between these markers and DKD, while receiver operating characteristic (ROC) curve analysis evaluated their predictive performance. Restricted cubic spline (RCS) models explored dose-response relationships, supplemented by subgroup and interaction analyses.

**Results:**

Higher quartiles of TyG, TyG-BMI, and TG/HDL-C were significantly associated with increased DKD risk (trend *P* < 0.001). ROC analysis revealed moderate-to-strong predictive accuracy for all three markers (AUC > 0.7). RCS modeling indicated a linear relationship between TyG and DKD risk (nonlinearity *P* = 0.378), whereas TyG-BMI and TG/HDL-C exhibited nonlinear associations (nonlinearity *P* < 0.05). Subgroup analysis identified a significant gender interaction for TG/HDL-C, with a stronger association in males (interaction *P* < 0.05). Age did not significantly modify these relationships. The biomarkers’ association with DKD was more pronounced in patients with HbA1c ≥ 7%, while the TyG-BMI–DKD link was weaker in those with HbA1c < 7%. Stratified analysis by BMI showed a significant interaction (interaction *P* < 0.05).

**Conclusion:**

TyG, TyG-BMI, and TG/HDL-C are positively correlated with DKD risk in T2DM patients and demonstrate substantial predictive value, supporting their potential as accessible, cost-effective indicators for DKD risk assessment.

## Introduction

Type 2 diabetes mellitus (T2DM) represents a complex metabolic disorder arising from insulin resistance (IR) in peripheral tissues coupled with pancreatic β-cell dysfunction, resulting in chronic hyperglycemia. This condition constitutes 90-95% of global diabetes cases (1). Among its most consequential microvascular complications, diabetic kidney disease (DKD) emerges as a progressive condition marked by deteriorating glomerular function. The initial phase features subtle renal hemodynamic alterations—including elevated pressure, perfusion, and filtration rates—while remaining clinically silent. As the disease advances, persistent proteinuria and declining glomerular filtration rate become hallmark features (2). Without intervention, DKD frequently progresses to end-stage renal disease (ESRD), necessitating renal replacement therapy. Current epidemiological data reveal that nearly 40% of diabetic patients develop DKD (3). The global burden of diabetes-associated ESRD has risen dramatically, increasing from 20% during the 20th century to 29.7% in recent years. Notably, in the United States, diabetes underlies nearly half of all ESRD cases (4). Beyond renal outcomes, DKD substantially elevates cardiovascular risk and all-cause mortality while imposing significant healthcare costs. These clinical and economic impacts underscore the urgent need for reliable, non-invasive predictive biomarkers to facilitate early DKD risk stratification.

IR refers to the reduced sensitivity and responsiveness of target organs such as the liver and muscles to insulin (5), which is the core pathogenic mechanism of T2DM, fatty liver and other diseases. Accumulating evidence indicates that IR not only accelerates DKD progression (6), but also persists in chronic kidney disease (CKD) patients, exacerbating cardiovascular mortality. Consequently, early IR detection and intervention in T2DM are critical for mitigating DKD advancement and improving clinical outcomes.

The gold standard for diagnosing IR (hyperinsulinemic-euglycemic clamp method) is complex and only suitable for small sample studies; the homeostasis model (HOMA-IR) is limited by insulin treatment and lacks a unified detection standard. Therefore, there is an urgent need for low-cost and easy-to-operate alternative indicators in clinical practice. Recent studies highlight lipid-derived indices—including the triglyceride-glucose (TyG) index, TyG-body mass index (TyG-BMI), and triglyceride-to-HDL cholesterol ratio (TG/HDL-C)—as promising alternatives (7, 8).

The TyG index, derived from fasting triglycerides and glucose, demonstrates high diagnostic accuracy for IR (9), and independently predicts DKD risk, correlating strongly with microalbuminuria (10). Studies have shown that combining obesity-related indicators (such as BMI) with the TyG index can significantly improve the assessment efficacy of IR (11), among which the TyG-BMI index is more predictive of IR than the TyG index, BMI, blood lipids, visceral obesity index, and lipid accumulation products (12, 13). Similarly, the TG/HDL-C ratio surpasses isolated lipid parameters in forecasting both IR and cardiovascular risk (14).

Despite these advances, the relationship between IR surrogates and DKD remains underexplored. This study evaluates the predictive utility of TyG, TyG-BMI, and TG/HDL-C for DKD, aiming to facilitate early diagnosis, reduce dialysis dependence, and alleviate the socioeconomic burden of diabetes complications.

## Materials and methods

### Study design and patient cohort

This hospital-based cross-sectional study evaluated 311 consecutive patients meeting WHO 1999 diagnostic criteria for T2DM (15), from the Jiangjin Hospital, Chongqing University of Chinese Medicine between January 2024 and April 2025. The exclusion criteria are as follows (1): Diagnosed with type 1 diabetes or other diabetes classified into specific types, such as latent autoimmune diabetes in adults (LADA); (2) Acute diabetic metabolic disorders: diabetic ketoacidosis (DKA), hyperosmolar hyperglycemic state (HHS), or other acute diabetic metabolic disorders necessitating urgent intervention (e.g., recurrent severe hypoglycemic episodes); (3) Presence of primary kidney disease, hypertensive nephropathy, and vascular self - deformity lesions; (4) Diagnosis of acute and chronic infections or neoplasms; (5) Renal failure, with an eGFR ≤ 15 mL/min; (6) Administration of nephrotoxic drugs or immunosuppressive agents within the past two weeks; (7) Use of lipid - lowering medications, such as statins and fibrates, within the past three months; (8) Those with incomplete data and information. The study was approved by the ethics committee at the authors’ hospitals (20230920).

### Clinical data collection

Demographic and laboratory parameters were systematically extracted from electronic medical records within 24 hours of admission. Collected variables included (1): anthropometric measures (age, sex, height, weight) and hemodynamic parameters (systolic/diastolic blood pressure) (2); hematologic indices: white blood cell count (WBC), platelet count (PTL), prothrombin time (PT), activated partial thromboplastin time (APTT), prothrombin time activity (PTA) (3); metabolic profiles: glycated hemoglobin (HbA1c), random glucose (Gluc), lipid panel including total cholesterol (TC), triglycerides (TG), and high-density lipoprotein cholesterol (HDL-L) (4); hepatic/renal function markers: alanine aminotransferase (ALT), aspartate aminotransferase (AST), total bilirubin (TBIL), creatinine (CREA), uric acid (UA), estimated glomerular filtration rate (eGFR); and (5) muscle injury indicators: creatine kinase (CK), lactate dehydrogenase (LDH). Post-stabilization oral glucose tolerance test results provided 0-hour glucose and C-peptide values. To ensure data integrity, dual independent extraction with cross-verification was implemented, with missing parameters rectified through clinical record review. Incomplete cases were excluded from final analysis to maintain dataset completeness.

### Clinical parameter calculation

The reference formula is as follows (16)::BMI = weight (kg)/height² (m²); TyG index = ln(FPG (mg/dL) × TG (mg/dL))/2; TyG-BMI = ln(FPG (mg/dL) × TG (mg/dL))/2 × BMI (kg/m2); TG/HDL-C = TG (mmol/L)/HDL-C (mmol/L).

### Diagnostic criteria for DKD

DKD was diagnosed according to established clinical criteria, requiring persistent albuminuria (urine albumin-to-creatinine ratio [UACR] >30 mg/g) for ≥3 months after exclusion of alternative etiologies including primary glomerulopathies and systemic disorders (17). Study participants with type 2 DM were stratified into three clinically relevant subgroups based on UACR measurements (1): normoalbuminuric (UACR < 30 mg/g, and eGFR ≥ 60 mL/min/1.73m²) (2), microalbuminuric (30–300 mg/g), and (3) macroalbuminuric (≥ 300 mg/g). For analytical purposes, the micro- and macroalbuminuric subgroups were collectively designated as the DKD cohort, while normoalbuminuric subjects comprised the non-DKD control group.

### Statistical analysis

All analyses were conducted using R (v4.2.2), SPSS (v27.0), and GraphPad Prism (v10.1.2). Data normality was assessed using the Kolmogorov-Smirnov test. Normally distributed continuous variables are presented as mean ± SD; non-normal variables as median (IQR). Categorical variables are expressed as percentages. Group comparisons employed ANOVA or χ² tests for normal data, and Kruskal-Wallis tests otherwise. Participants were stratified by quartiles (Q1-Q4) of IR surrogate markers. Multivariable regression models evaluated DKD associations: Model 1 (unadjusted); Model 2 (adjusted for age/sex); Model 3 additional adjustments for BMI, blood pressure, and comprehensive laboratory parameters (WBC, PLT, PT, PTA, APTT, HbA1c, Gluc, TC, TG, HDL-L, ALT, ALB, TBIL, CREA, UA, CK, LDH, 0-h glucose, and 0-h C-peptide). The predictive value of IR surrogate markers for DKD was analyzed using the receiver operating characteristic (ROC) curve and the area under the curve (AUC). Additionally, the restricted cubic spline (RCS) method was used to explore the potential nonlinear relationship between IR surrogate markers and DKD. To ensure the robustness of the study results, subgroup analyses were conducted by stratifying the population by gender, age, and glycated hemoglobin levels, and the *P* values for interaction were calculated to assess the differences among subgroups. All statistical analyses were two-sided, and a *P* value < 0.05 was considered statistically significant.

## Results

### Baseline characteristics

**Figure 1:**
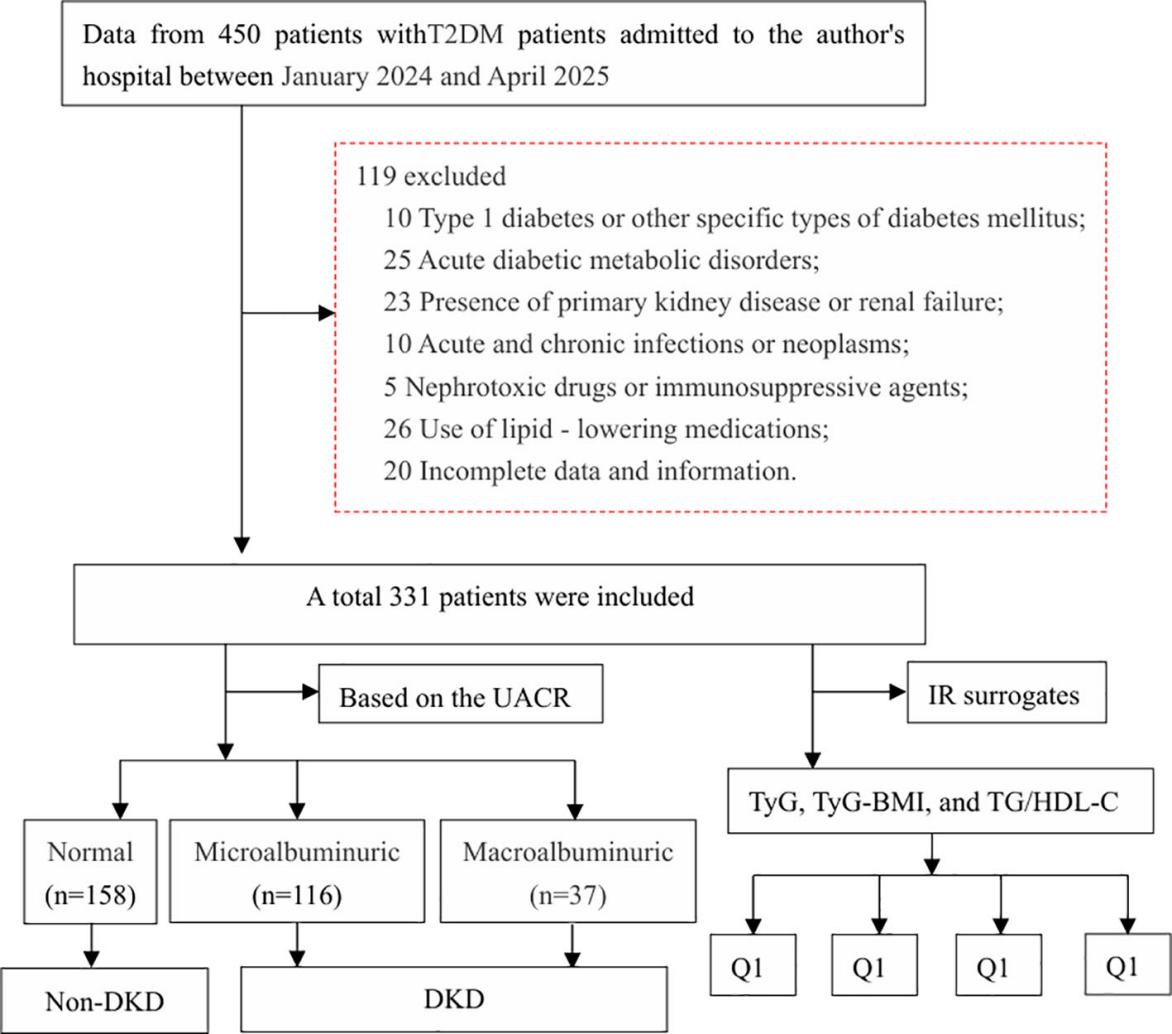
Flow chart of patient selection.

**Figure 2:**
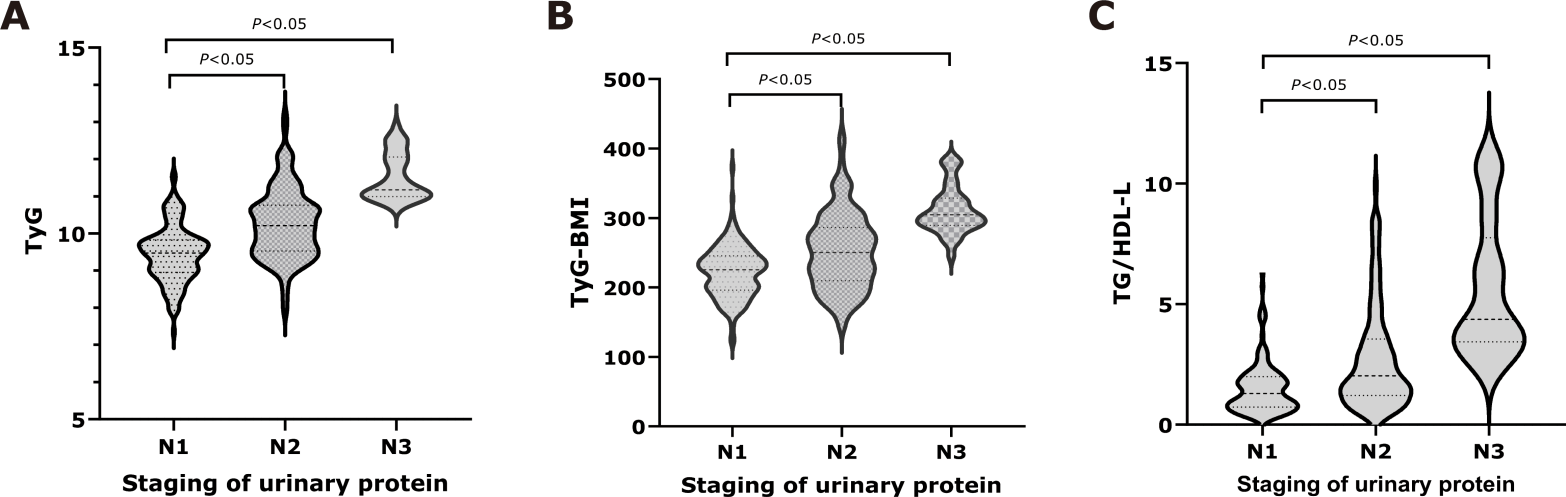
Comparison of IR surrogates among three groups. Normoalbuminuria group (N1), microalbuminuria group (N2), and macroalbuminuria group (N3). **(A)** for TyG, **(B)** for TyG-BMI, and **(C)** for TG/HDL-C.

The final cohort comprised 311 rigorously selected T2DM patients (**Figure 1**), stratified by albuminuria status: normoalbuminuric (n=158, 50.8%), microalbuminuric (n=116, 37.3%), and macroalbuminuric (n=37, 11.9%). Compared to normoalbuminuric controls, both albuminuric groups demonstrated significantly elevated BMI, HbA1c, fasting glucose, triglycerides, creatinine, and uric acid levels; conversely, the eGFR level was significantly reduced (all *P* < 0.05, **Table 1**). Insulin resistance surrogate markers (TyG, TyG-BMI, TG/HDL-C) showed progressive elevation across worsening albuminuria categories (*P* < 0.05, **Figure 2**).

**Table 1:** Comparison of baseline data among different proteinuria conditions in patients with T2DM.

Characteristic	Overall N = 311	Normal N = 158	Microalbuminuria N = 116	Macroalbuminuria N = 37	*P*-value
Demographics					
Sex, n (%)					0.169
Female	140 (45.0%)	66 (41.8%)	60 (51.7%)	14 (37.8%)	
Male	171 (55.0%)	92 (58.2%)	56 (48.3%)	23 (62.2%)	
Age (year)	58 (51, 66)	58 (51, 67)	58 (51, 66)	58 (53, 62)	0.994
SBP (mmHg)	131 (120, 145)	130 (119, 142)	132 (118, 148)	136 (126, 156)	0.053
DBP (mmHg)	82 (74, 93)	83 (74, 93)	81 (75, 92)	87 (75, 94)	0.378
BMI (kg/m²)	24.2 (21.6, 26.5)	23.4 (21.4, 25.4)	24.2 (21.4, 26.9)	26.7 (25.5, 28.7)	<0.001
Laboratory indices					
WBC (×10^9^/L)	6.70 (5.53, 8.33)	6.51 (5.40, 8.18)	6.74 (5.61, 8.32)	6.85 (5.94, 9.57)	0.176
PLT (×10^9^/L)	203 (171, 250)	201 (173, 248)	208 (169, 254)	210 (171, 259)	0.944
PT (Sec)	12.70 (12.30, 13.40)	12.80 (12.30, 13.50)	12.90 (12.30, 13.40)	12.40 (12.20, 12.90)	0.084
APTT (Sec)	26.7 (24.2, 28.8)	27.4 (24.6, 29.1)	26.2 (23.6, 28.4)	25.9 (23.6, 27.8)	0.057
HbA1c (%)	10.70 (8.60, 12.10)	10.40 (8.10, 11.90)	10.80 (9.05, 12.00)	11.10 (10.10, 13.20)	0.019
Gluc (mmol/l)	11.8 (8.9, 16.5)	10.1 (8.1, 12.5)	13.9 (10.4, 17.8)	21.6 (16.1, 25.8)	<0.001
TC (mmol/l)	5.07 (4.37, 6.05)	4.93 (4.32, 5.89)	5.32 (4.36, 6.51)	5.20 (4.63, 6.05)	0.375
TG (mmol/l)	2.1 (1.2, 3.8)	1.5 (0.9, 2.2)	2.6 (1.4, 4.1)	5.1 (4.1, 11.0)	<0.001
HDL-L (mmol/l)	1.27 (1.06, 1.52)	1.24 (1.06, 1.53)	1.31 (1.07, 1.53)	1.27 (1.11, 1.49)	0.690
ALT (U/L)	20 (14, 34)	19 (15, 35)	22 (14, 36)	17 (12, 27)	0.202
AST (U/L)	20 (16, 28)	21 (16, 27)	21 (18, 32)	17 (15, 24)	0.056
TBIL (μmol/L)	13 (10, 16)	13 (10, 16)	13 (10, 17)	11 (9, 15)	0.313
CREA (μmol/L)	60 (50, 73)	59 (49, 70)	60 (51, 75)	81 (59, 104)	<0.001
eGFR (mL/min/1.73m²)	102 (89, 111)	104 (95, 112)	101 (88, 113)	95 (75, 106)	0.002
UA (μmol/L)	285 (221, 364)	269 (213, 340)	290 (221, 378)	323 (287, 398)	<0.001
CK (U/L)	71 (50, 98)	72 (50, 95)	68 (49, 96)	75 (55, 121)	0.343
LDH (U/L)	144 (125, 181)	142 (122, 170)	145 (125, 177)	161 (138, 205)	0.057
0-h glucose (mmol/l)	7.81 (6.47, 9.19)	7.67 (6.22, 9.11)	8.09 (6.64, 9.33)	7.74 (6.65, 9.12)	0.588
0-h C-peptide (ng/ml)	0.89 (0.54, 1.56)	0.94 (0.54, 1.65)	0.83 (0.52, 1.46)	0.85 (0.62, 1.77)	0.632
Insulin resistance surrogates					
TyG	9.80 (9.26, 10.74)	9.47 (8.95, 9.82)	10.21 (9.53, 10.76)	11.18 (10.99, 12.05)	<0.001
TyG-BMI	238 (205, 276)	226 (196, 245)	250 (211, 286)	305 (289, 327)	<0.001
TG/HDL-C	1.74 (0.93, 3.17)	1.30 (0.73, 1.99)	2.03 (1.21, 3.54)	4.37 (3.44, 7.11)	<0.001

SBP, systolic and diastolic blood pressure; DBP, diastolic blood pressure; BMI, body mass index; WBC, white blood cell count; PLT, platelet; PT, prothrombin time; APTT, activated partial thromboplastin time; HbA1c, glycated hemoglobin a1c; Gluc, glucose; TC, total cholesterol; TG, Triglyceride; HDL-L, high-density lipoprotein; ALT, alanine aminotransferase; AST, aspartate aminotransferase; TBIL, total bilirubin; CREA, creatinine; eGFR, estimated glomerular filtration rate; UA, uric acid; CK, creatine kinase; LDH, lactate dehydrogenase; TyG, triglyceride-glucose index; TyG-BMI, triglyceride-glucose-body mass index; TG/HDL-C, triglyceride to high-density lipoprotein cholesterol ratio.

### Correlation between quartiles of IR surrogate markers and DKD

Patients with T2DM were stratified into four groups according to quartiles of IR surrogate markers. Multivariate logistic regression analysis (**Table 2**) revealed that the risk of DKD increased significantly with increasing quartiles of TyG, TyG-BMI, and TG/HDL-C (trend test *P* < 0.001). In Model 1, without adjustment for any variables, TyG, TyG-BMI, and TG/HDL-C exhibited significant associations with DKD (all *P* < 0.05). In Model 2, following adjustment for sex and age, all IR surrogate markers remained significantly associated with DKD (all *P* < 0.05). In Model 3, following further comprehensive adjustment for metabolic and clinical covariates, the highest quartiles of these markers retained significant associations with DKD (all *P* < 0.05). DKD risk was highest in the fourth quartile (Q4) for all markers, with odds ratios (ORs) of 28.37 (95% confidence interval (CI): 8.50–94.68) for TyG, 92.08 (95% CI: 13.33–636.03) for TyG-BMI, and 14.91 (95% CI: 2.07–107.60) for TG/HDL-C.

**Table 2:** Multivariate logistic regression analysis of IR substitutes for DKD.

Characteristic	Model 1		Model 2		Model 3	
	OR (95% CI)	*P*-value	OR (95% CI)	*P*-value	OR (95% CI)	*P*-value
TyG						
Q1	Reference		Reference		Reference	
Q2	2.29 (1.08, 4.82)	0.030	2.25 (1.06, 4.78)	0.035	1.94 (0.82, 4.59)	0.130
Q3	6.43 (3.08, 13.40)	<0.001	7.02 (3.31, 14.89)	<0.001	6.11 (2.40, 15.55)	<0.001
Q4	31.09 (12.89, 74.97)	<0.001	38.61 (15.38, 96.96)	<0.001	28.37 (8.50, 94.68)	<0.001
TyG-BMI						
Q1	Reference		Reference		Reference	
Q2	0.94 (0.48, 1.84)	0.864	0.94 (0.48, 1.86)	0.870	1.92 (0.75, 4.94)	0.177
Q3	1.42 (0.74, 2.73)	0.291	1.53 (0.78, 2.97)	0.213	4.09 (1.26, 13.32)	0.019
Q4	17.50 (7.33, 41.76)	<0.001	22.41 (9.02, 55.71)	<0.001	92.08 (13.33, 636.03)	<0.001
TG/HDL-C						
Q1	Reference		Reference		Reference	
Q2	1.89 (0.96, 3.70)	0.064	2.03 (1.02, 4.01)	0.043	4.62 (1.72, 12.38)	0.002
Q3	2.26 (1.16, 4.43)	0.017	2.63 (1.32, 5.26)	0.006	4.26 (1.29, 14.04)	0.017
Q4	13.57 (6.24, 29.54)	<0.001	18.84 (8.13, 43.66)	<0.001	14.91 (2.07, 107.60)	0.007

Model 1. no covariates were adjusted.

Model 2. adjusted for Sex and Age.

Model 3. adjusted for Sex, Age, SBP, DBP, BMI, WBC, PLT, PT, PTA, APTT, HbA1c, Gluc, TC, TG, HDL-L, ALT, ALB, TBIL, CREA, UA, CK, LDH, 0-h glucose, and 0-h C-peptide.

### Predictive value of IR surrogate markers for DKD

**Figure 3:**
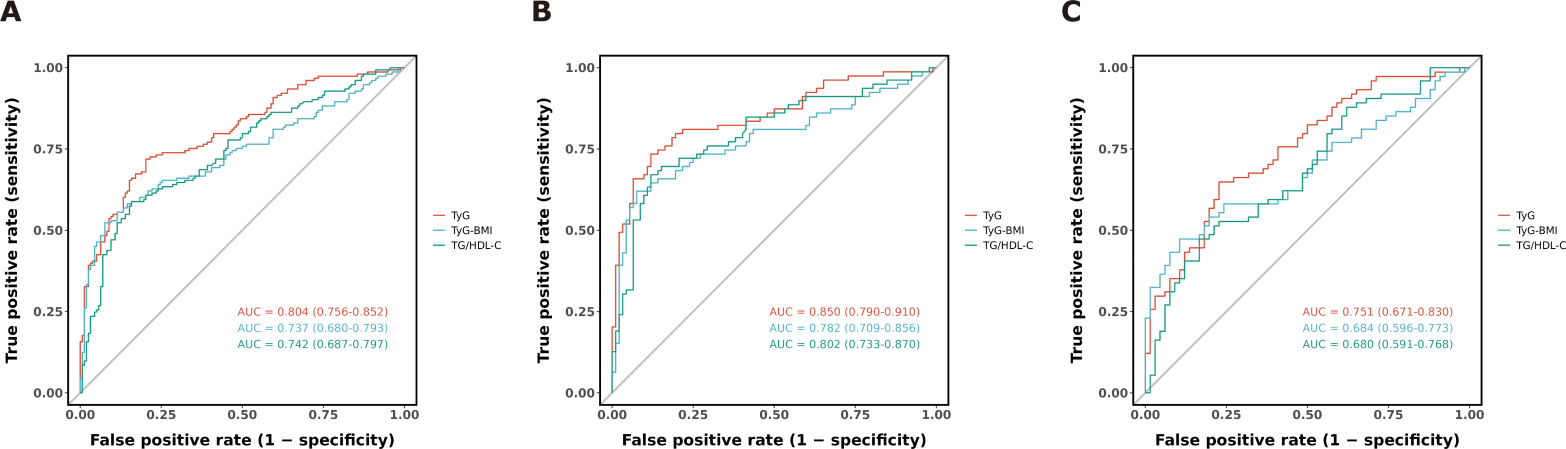
The ROC curve of IR surrogate indicators for DKD in T2DM. **(A)** represents the entire population, **(B)** represents male, and **(C)** represents female.

Assessed the utility of IR surrogate markers in predicting DKD among patients with T2DM via ROC curve analysis. Detailed metrics including AUC, 95% CI, optimal threshold values, Youden indices, and corresponding sensitivity and specificity are provided in **Table 3**. In the total cohort, TyG, TyG-BMI, and TG/HDL-C all exhibited significant discriminative power for identifying T2DM patients with DKD, with AUCs exceeding 0.7, indicating moderate to high discriminative capacity. Gender-stratified ROC analyses (**Figure 3**) further revealed that among males, these markers showed higher AUCs for DKD discrimination, with TyG achieving the highest (0.850, 95% CI 0.790–0.910). Among females, all IR surrogates similarly demonstrated high AUCs and sensitivity, with TyG again yielding the highest AUC (0.751, 95% CI 0.671–0.830).

**Table 3:** Surrogate markers of IR for predicting DKD.

Characteristic	AUC (95% CI)	Cut point	Youden	Sensitivity	Specificity	Accuracy
Total						
TyG	0.804 (0.756-0.852)	9.908	0.516	71.9%	79.7%	75.9%
TyG-BMI	0.737 (0.680-0.793)	268.604	0.447	52.3%	92.4%	72.7%
TG/HDL-L	0.742 (0.687-0.797)	2.271	0.430	58.8%	84.2%	71.7%
Males						
TyG	0.850 (0.790-0.910)	10.167	0.615	73.4%	88.0%	81.3%
TyG-BMI	0.782 (0.709-0.856)	264.468	0.544	62.0%	92.4%	78.4%
TG/HDL-L	0.802 (0.733-0.870)	2.455	0.551	67.1%	88.0%	78.4%
Females						
TyG	0.751 (0.671-0.830)	9.908	0.421	64.9%	77.3%	70.7%
TyG-BMI	0.684 (0.596-0.773)	268.604	0.367	47.3%	89.4%	67.1%
TG/HDL-L	0.680 (0.591-0.768)	2.271	0.306	47.3%	83.3%	64.3%

### RCS analysis of IR surrogate markers and DKD in T2DM patients

**Figure 4:**
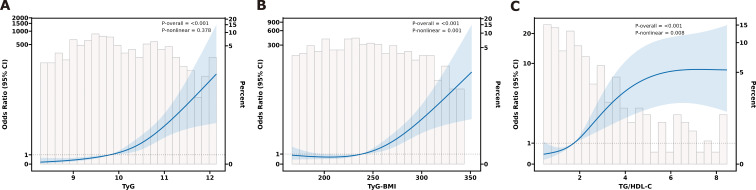
The dose-response relationship between IR surrogate indicators and DKD risk. **(A)** for TyG, **(B)** for TyG-BMI, and **(C)** for TG/HDL-C.

The dose-response relationship between IR surrogate markers and the risk of DKD was further explored using the RCS regression model. The RCS curves after adjusting for confounding factors (**Figure 4**) showed that TyG was linearly related to DKD (*P*-nonlinear = 0.378); TyG-BMI and TG/HDL-C were nonlinearly related to DKD (both *P*-nonlinear < 0.05).

### Association of IR surrogate markers with DKD and stratified interaction analysis

**Figure 5:**
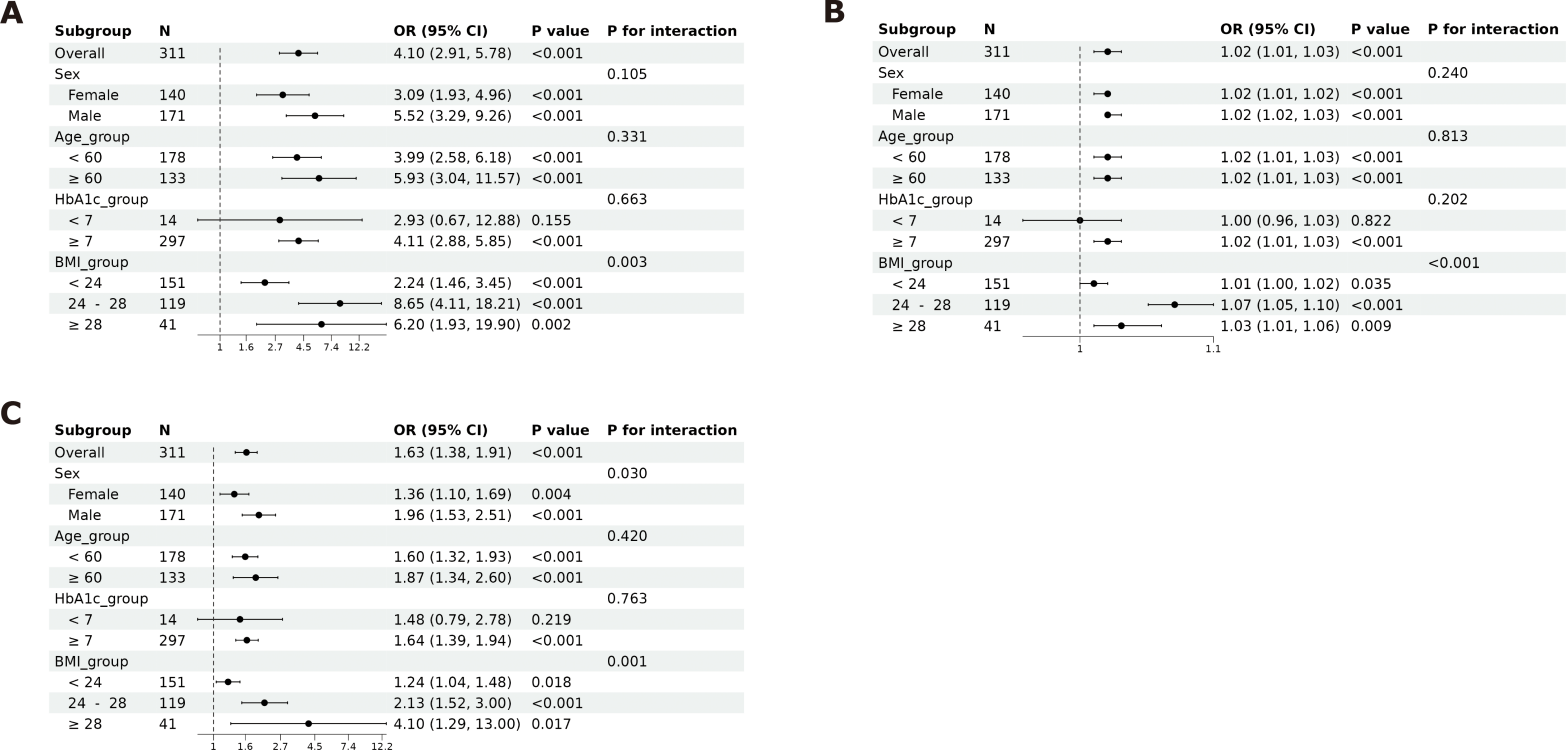
Correlation and hierarchical interaction analysis between IR surrogate indicators and DKD.

After adjustment for potential confounders including age, sex, and glycated hemoglobin levels, we performed stratified and interaction analyses to characterize associations between IR surrogate markers and DKD. Key findings are as follows:

**Figure 5A** demonstrates that TyG is significantly and positively associated with DKD risk in the overall cohort (OR = 4.10, 95% CI: 2.91–5.78, *P* < 0.001). Subgroup analyses revealed no significant interactions with sex (*P* for interaction = 0.105) or age (*P* for interaction = 0.331). The association was slightly stronger in male than in female (ORs 5.52 vs 3.09) and in participants aged ≥60 years compared with those <60 years (ORs 5.93 vs 3.99). While no significant interaction was observed across HbA1c strata (*P* for interaction = 0.663), the association remained significant only in the HbA1c ≥ 7% subgroup (indicating poor glycemic control; *P* < 0.001). Stratified analyses based on BMI revealed a statistically significant interaction (*P* for interaction = 0.003). The strength of the association varied substantially across BMI categories: it was most pronounced in the overweight group (BMI 24–28 kg/m²; OR = 8.65, 95% CI: 4.11–18.21, *P* < 0.001), followed by the obese group (BMI ≥ 28 kg/m²; OR = 6.20, 95% CI: 1.93–19.90, *P* = 0.002), and was more modest in the normal-weight group (BMI < 24 kg/m²; OR = 2.24, 95% CI: 1.46–3.45, *P* < 0.001).

**Figure 5B** shows that TyG-BMI is significantly and positively correlated with DKD risk in the total population (*P* < 0.001). Subgroup analyses confirmed consistent associations across sex and age strata, though the relationship may lack statistical significance in the HbA1c < 7% subgroup (indicating good glycemic control). Stratified analysis by BMI revealed a significant interaction effect (*P* for interaction < 0.001). The magnitude of risk elevation followed a gradient across BMI categories, being most substantial in the overweight group (BMI 24–28 kg/m²; OR = 1.07, *P* < 0.001), intermediate in the obese group (BMI ≥ 28 kg/m²; OR = 1.03, 95%, *P* = 0.009), and relatively modest in the normal-weight group (BMI < 24 kg/m²; OR = 1.01, *P* = 0.035).

**Figure 5C** reveals that TG/HDL-C is significantly and positively associated with DKD risk in the overall cohort (*P* < 0.001). Subgroup analyses identified a significant interaction with sex (*P* for interaction < 0.05), with a stronger association in male (OR = 1.96). No significant interaction was observed with age. While associations varied by HbA1c level (without reaching statistical significance for interaction), the relationship was more pronounced in the HbA1c ≥ 7% subgroup (*P* < 0.001). With the increase in BMI, the association strength between TG/HDL-C and the risk of diabetic nephropathy significantly enhanced (BMI < 24 kg/m²: OR = 1.24, *P* = 0.018; BMI 24–28 kg/m²: OR = 2.13, *P* < 0.001; BMI ≥ 28 kg/m²: OR = 4.10, *P* = 0.017).

## Discussion

This study sought to assess the association between IR surrogate markers and DKD in patients with T2DM, as well as their predictive value for DKD. Consistent with these aims, our key findings are as follows (1): Compared with the normoalbuminuria group, patients in the albuminuria-positive group exhibited derangements in glucose and lipid metabolism, suboptimal glycemic control, and more pronounced IR (*P* < 0.05) (2). Multivariate logistic regression analyses revealed that DKD risk rose significantly with increasing quartiles of TyG, TyG-BMI, and TG/HDL-C (*P* for trend < 0.001) (3). ROC curve analyses demonstrated that all three markers had an AUC > 0.7 (4). RCS analyses indicated a linear relationship between TyG and DKD risk (*P*-nonlinear = 0.378), whereas TyG-BMI and TG/HDL-C showed non-linear associations (*P*-nonlinear < 0.05) (5). Subgroup analyses revealed that sex exerted a significant interaction effect on the association between TG/HDL-C and DKD, with a stronger association in males (*P* for interaction < 0.05). No significant interaction was observed with age. The associations of all three markers with DKD were more pronounced in the HbA1c ≥ 7% subgroup, while the association of TyG-BMI with DKD may lack statistical significance in the HbA1c < 7% subgroup. Stratified analysis by BMI showed a significant interaction (interaction *P* < 0.05).

Our findings reveal that patients with DKD exhibit more severe clinicopathological features, including dysregulated glucose and lipid metabolism, inadequate glycemic control, and pronounced IR (**Table 1**, **Figure 2**). The onset and progression of DKD are tightly linked to disturbances in glucose and lipid homeostasis, as well as IR. Additionally, uric acid levels were elevated in DKD patients in our cohort. Prior studies have similarly demonstrated that hyperuricemia exacerbates renal injury through mechanisms such as inducing oxidative stress and triggering inflammatory responses (18). Clinical investigations have established that optimal glycemic control significantly reduces the incidence of proteinuria, effectively slows the rate of renal function decline, and thereby delays DKD progression (19). A study by Huang et al. noted that TG and TC levels in DKD patients were significantly higher than in healthy controls, with values positively correlated with the severity of renal dysfunction (20). Accumulating evidence indicates a significant positive association between IR and the development of DKD; potential mechanisms by which IR contributes to DKD progression include renal functional impairment via induction of oxidative stress, release of inflammatory factors, and disruption of insulin signaling pathways, collectively accelerating renal deterioration (21). These findings align with our observations.

Here, this cross-sectional study systematically assessed the predictive value of three IR surrogate markers for DKD risk in individuals with T2DM. Multivariate logistic regression analyses revealed that DKD risk in T2DM patients rose with increasing quartiles of these IR surrogates. Following adjustment for confounding factors including sex, age, blood pressure, BMI, and laboratory parameters, the highest quartiles of TyG, TyG-BMI, and TG/HDL-C remained independently associated with DKD risk. ROC curve analyses demonstrated that TyG, TyG-BMI, and TG/HDL-C conferred predictive value for DKD in both male and female subgroups, supporting their potential as simple, cost-effective, and clinically feasible markers for predicting DKD onset in T2DM patients. Notably, previous studies have stratified the TyG index into tertiles, reporting that the highest tertile correlates positively with microalbuminuria prevalence, with an adjusted OR of 1.727 (95% CI 1.103–2.703) (22). A multicenter study across eight regions in China identified a non-linear association between the TG/HDL-C ratio and microalbuminuria, with an 89% higher risk of microalbuminuria per unit increase in TG/HDL-C below the inflection point, and a 12% higher risk above this threshold (23). Our findings align with these observations, though no prior research has examined the association between the TyG-BMI index and DKD onset or progression.

Dysregulated glucose and lipid metabolism represent key hallmarks of IR, and the pathogenesis of DKD is intricately linked to such metabolic perturbations. Specifically, dyslipidemia and hyperglycemia act in concert to drive the development and progression of diabetic nephropathy. Notably, hyperglycemia induces renal injury through multiple pathways, including the accumulation of advanced glycation end products (AGEs), mitochondrial dysfunction, and altered renal hemodynamics, among others (24). These pathological cascades culminate in the hallmark lesions of DKD, such as glomerulosclerosis and tubulointerstitial fibrosis (25). Sustained hyperglycemia further amplifies oxidative stress, triggers inflammatory responses and cell death, and establishes a “metabolic memory” phenomenon, thereby exacerbating DKD progression (26). Prior research has established that optimal glycemic control significantly reduces proteinuria, slows the decline in renal function, and thereby retards DKD progression (19). Our findings revealed that the associations of TyG, TyG-BMI, and TG/HDL-C with DKD were more pronounced in the HbA1c ≥ 7% subgroup, suggesting that suboptimal glycemic control may exacerbate renal injury and elevate DKD risk.

The mechanisms underlying lipid dysregulation in DKD are complex and multifactorial, with lipotoxicity emerging as a critical driver of disease progression. Aberrant lipid accumulation triggers inflammatory responses, enhances free radical generation, and induces renal cellular damage (27, 28). Specifically, lipid disorders promote inflammatory cell infiltration and the release of pro-inflammatory cytokines by activating signaling pathways such as LOX-1/NF-κB (29). Concurrently, lipid abnormalities induce mitochondrial oxidative stress, impair energy metabolism, and drive excessive production of reactive oxygen species (ROS), leading to podocyte apoptosis and glomerular basement membrane injury (30). Furthermore, lipid overload disrupts podocyte cytoskeletal architecture and increases glomerular permeability through the regulation of key factors including RhoA and Rac1 (31). Epidemiological evidence indicates that each 0.5 mmol/L increase in TG levels is associated with a 23% elevation in DKD risk (32), while studies in Chinese populations have confirmed that controlling TG levels can slow the decline of early renal function (33). These findings underscore the pivotal role of lipid dysregulation in DKD pathogenesis, aligning with the observations of our study.

Notably, derangements in glycolipid metabolism and IR reinforce one another, perpetuating a vicious cycle of “metabolic dysfunction–IR–renal injury”. IR drives DKD progression via multiple mechanisms, including the amplification of inflammatory responses, oxidative stress, endothelial dysfunction, and extracellular matrix deposition, among others (16). Furthermore, hyperinsulinemia in the setting of IR may drive renal cell proliferation and fibrosis by activating signaling cascades such as JAK/STAT, MAPK, mTOR, Wnt/β-catenin, and PI3K/Akt (34, 35). Studies have demonstrated that the nucleotide-binding oligomerization domain 2 (NOD2) receptor not only couples inflammation to podocyte dysfunction (36), but also impairs insulin signaling in podocytes, thereby exacerbating renal damage (37). Clinical investigations have established that the severity of IR correlates strongly with increased microalbuminuria and a marked decline in eGFR in diabetic individuals (38), offering new insights into the pathogenesis of DKD.

The TyG index, first introduced by researchers including SM (39), is a surrogate marker for IR calculated using fasting glucose and triglyceride levels. Evidence indicates that, compared with HOMA-IR, the TyG index exhibits greater sensitivity and specificity in identifying IR (40). Studies in the US population have confirmed that an elevated TyG index is independently associated with albuminuria (41) —findings consistent with our results, which not only validate the TyG index’s predictive value for DKD in T2DM patients but also underscore the critical role of glycolipotoxicity in DKD progression. In this study, the exclusion of patients on lipid-lowering therapy ensures that the observed elevations in the TyG index, particularly in the macroalbuminuria group, reflect the inherent dysmetabolic state associated with advanced DKD rather than being influenced by pharmacologic modulation. Given that obesity is a key driver of IR, the TyG-BMI index, by integrating BMI, may more comprehensively reflect IR status. Our study identified a stable nonlinear association between TyG-BMI and DKD, consistent across sex and age subgroups. Notably, this association appeared nonsignificant in the well-controlled glycemia subgroup (HbA1c < 7%). Currently, research on TyG-BMI and DKD remains limited, warranting large-scale validation. While prior studies suggest TyG-BMI outperforms TyG, TyG-WC, and TyG-WHtR in predicting IR (8), another study found the TyG index to be more robust than TyG-BMI in predicting diabetes and its complication DKD (42) —a conclusion aligned with our findings. ROC analyses in both the overall cohort and sex-stratified subgroups indicated the TyG index has higher predictive value for DKD than TyG-BMI, potentially due to limitations of BMI: although it identifies obesity, it fails to distinguish between fat distribution patterns and their metabolic activity. Elevated TG and reduced HDL-C are hallmarks of metabolic dysfunction, and their ratio (TG/HDL-C) serves as a marker of metabolic derangement severity. An increased TG/HDL-C ratio may accelerate DKD progression by inhibiting insulin signaling, promoting adipogenesis, and triggering pro-inflammatory factor release (43). A prior study in Shanghai community populations found the dose-response relationship between TG/HDL-C and DKD risk peaked at a ratio of 2.0 (44), close to the ROC-defined cutoff of 2.271 in our study. Such cutoff differences may stem from variations in insulin sensitivity and detection standards across regions and ethnicities. Notably, the association between TG/HDL-C and DKD is stronger in male patients—likely linked to men’s greater propensity for visceral fat accumulation, smoking, and alcohol consumption, which synergistically exacerbate renal damage—highlighting the need to prioritize lipid management in male T2DM patients.

Several limitations should be considered when interpreting our findings. First, the single-center design and modest sample size may introduce selection bias and limit the generalizability of results across diverse populations and geographic regions, necessitating validation through multicenter studies with larger cohorts. Second, the cross-sectional nature of this analysis precludes causal inference regarding the observed associations between insulin resistance surrogates and diabetic kidney disease, while also preventing assessment of longitudinal dynamics in these relationships. Thirdly, although comprehensive multivariable adjustment was performed, residual confounding may persist due to unmeasured or unrecorded variables. These include lifestyle factors (e.g., dietary habits and physical activity levels), genetic predisposition, specific comorbidities, and concomitant medications influencing glucose metabolism. These limitations underscore the necessity for future prospective, population-based studies that incorporate detailed phenotypic characterization and systematic documentation of comorbidities and medication use to validate and extend the present findings. Furthermore, the TyG index is calculated from fasting parameters and thus does not reflect postprandial metabolic excursions. While the fasting index has proven to be a robust epidemiological tool, future studies incorporating dynamic tests, such as the TyG index during an oral glucose tolerance test, could provide a more comprehensive assessment of metabolic dysregulation and its relationship with DKD progression.

## Conclusion

This study systematically and jointly evaluated three IR surrogate markers, namely TyG, TyG-BMI, and TG/HDL-C, and found that they were significantly positively correlated with the risk of DKD in T2DM patients. Moreover, all three markers demonstrated good predictive value for DKD, and are expected to become simple and economical biological indicators for assessing the risk of DKD.

## Data Availability

The raw data supporting the conclusions of this article will be made available by the authors, without undue reservation.
